# Relationship between anatomical characteristics and personality traits in Lipizzan horses

**DOI:** 10.1038/s41598-022-16627-z

**Published:** 2022-07-23

**Authors:** Nataša Debeljak, Aljaž Košmerlj, Jordi Altimiras, Manja Zupan Šemrov

**Affiliations:** 1 Department of Animal Science, Biotechnical Faculty, University of Ljubljana, Groblje 3, 1230 Domžale, Slovenia; 2 Jozef Stefan Institute, Ljubljana, Slovenia; 3 Linköping University, Linköping, Sweden

**Keywords:** Musculoskeletal system, Cardiovascular biology, Psychology

## Abstract

We tested 35 Lipizzan horses older than 5 years, ridden and healthy in three behavioural tests (handling, fear-reaction, and target training test). Physiological (heart rate and heart rate variability) and anatomical measurements (120 head and body distances and angles) were collected to validate parameters that reliably inform on handling/cooperation, fear/exploration and trainability in horses. Utilizing a standard clustering methodology on the behavioural data, we identified four general types of responses and categorised an individual as intermediate, low fearful, horses with low cooperation or low trainability. We additionally analysed the head morphology following Tellington-Jones and Taylor recommendations and correlated the measurements with data from a horse personality questionnaire. Although allocation to a particular personality group was not associated with these two methods, these groups differed in six anatomical characteristics of head and body. Regardless of the group, our results indicated that shorter horses (<75.9 cm) with a wider muzzle (>10.5 cm) are trustworthy, less fearful and easier to handle and train. We also demonstrated that horses with stronger legs and a wider base of the head have a lower heart rate when exposed to the second trial of the handling test.

## Introduction

Horse owners and caretakers frequently provide anecdotal evidence supporting the existence of family trends in behaviour and temperament, which are aspects of the personality of a horse. Although horse personality assessment protocols have been developed, progress on assessing their reliability and optimizing their use has been slow. Horse breeders rely on the ability to select a horse that is trustworthy, explorative, easy to handle, relaxed and that does not show fearful responses or panic and there is evidence that personality traits can be used to select suitable training and weaning methods, choose or breed horses for police or therapeutic work, investigate underlying reasons for development of behavioural problems or assess how an unknown horse might react to a new or aversive situation or stimulus^1–4^.

Studies on horse personality typically rely on only one or two methods^5^ and a multifactorial approach when looking at individual behaviour differences over time and in different contexts is lacking. Four different methods are used for personality assessment of horses^5,6^: (1) questionnaire-based, (2) behavioural tests, (3) heart rate-based and (4) grading by anatomical characteristics. The first two methods have already seen extensive use, while the other two methods are still under development, particularly the last method where there are very limited findings, focused mainly on horse’s facial hair whorls^6–8^.

To our knowledge, the role of conformation (shape or structure of the horse) on personality has not been investigated. But horse conformation has been linked to biomechanics, and this in turn may affect personality characteristics^9,10^. Tellington-Jones and Taylor^9^ concluded that an ideal thoroughbred with great athletic abilities should have head, neck, shoulders, back and croup of the same length. Severe deviations in measurements, except for the croup, could cause stiffness and pain, which could cause unwillingness, unsoundness, and resistance^9,11^, although scientific evidence with objective data is lacking. A short back is thought to result in fewer back pain problems but more scalping problems than horses with a long back, and horses with higher withers and/or larger body are more prone to lameness problems. Groesel et al.^10^ showed that the length of the back muscle and consequently the length of the back affects horse movement.

An association between body characteristics and personality has been claimed for many species. The most excitable pigs and cattle have long slender bodies and fine bones^6^ and more dominant chimpanzees have a larger frontal cortex^12^. Holl et al.^13^ found that pigs and cattle with large bulging muscles often have calmer temperaments compared to lean animals with less muscle. In sheep, Hansen et al.^14^ observed that lighter breeds had stronger flocking behaviour and larger flight zones when confronted with threatening stimuli. Krushinskii and Haigh^15^ reported that slender, narrow-bodied dogs had increased excitability and were more fearful compared to “athletic wide-bodied” dogs. McGreevy et al.^16^ noted different behaviours that were linked with height, body weight or skull shape in dogs. One possible reason for the lack of literature on conformation scoring in horses, as mentioned by Back and Clayton^17^, is that current methods are suboptimal, with subjectively defined traits and no adequate information on their relative weights. Although Tellington-Jones and Taylor^9^ described a different number of characteristics of a horse’s head and linked them to specific horse personality traits, they were not consistently defined in any objective way and its specific meaning was not validated.

To choose the behavioural tests in our study, we evaluated what riders, breeders or owners consider important in a horse. According to the questionnaire of Graf et al.^3^, respondents assigned more importance to personality-related character and temperament traits than to performance traits. In another questionnaire-based study by Axel-Nilsson et al.^4^, participants marked the trait ‘ease to bring to new environments' as the most important. Our review of popular science resources indicated that confidence, cooperativity, and trainable traits are most wanted by horse trainers, breeders and riders.

Other characteristics known to be of a great importance are fear of objects, sounds, and movements^18^. The challenge can be even bigger for a horse, if these stimuli are combined: thus, the situation where a horse is faced with a moving unknown object that produces sound is considered highly challenging. With this in mind, we put together a battery of three behavioural tests: a handling test (HT^19^), a fear reaction test (FRT^20^), and a training test labelled a target training test (TTT^1,21^). The selected behavioural tests measure personality traits of curiosity^22^, ease of handling or willingness to cooperate^19,23^ in the HT, fearfulness in the FRT^20^, as well as trainability within a context in the TTT test (ungulates^24^).

To explore a comprehensive and non-invasive approach to robust phenotypic characterization in horses we used the Lipizzan horse as a model. This oldest cultural horse breed in the world^25^, whose current population is estimated at about 12,300 animals^26^, was chosen because its current breeding programme is based on descriptive and linear scoring methods and because evaluation is relative to the breeding objective. In this way, one only knows how close or far away the horse's phenotype appears to be from the breeding objective. Not only is this process subjective, but if a horse's phenotype is incorrectly evaluated, economic losses will result. The breed is characterised by longevity, excellent stamina, compact, elegant body, graceful movements, willingness to learn, good and strong temperament, courage and tenacity. These qualities make the breed suitable for classical dressage, but it is also used for other purposes. It can be an integral part of rituals, festive events and equestrian sports, and plays a special role in the cultural and social life of communities in rural areas^25^.

However, we still know little about how to recognise a good riding, working, companion or therapeutic Lipizzan horse at an early age. To address this lack of information, in this multifactorial study, three main objectives were considered: (1) assign individual horses to response groups based on different behaviour patterns; (2) evaluate variation in anatomy of head and body and cardiovascular activity of all horses and within the response group; (3) to evaluate objectivity of the used methods and identify the ones that are easy to implement under practical conditions.

## Results

### The relationship between anatomical and physiological measurements and horse behaviour

To analyse the relationship between horse physiology and behaviour in this pilot study, we decided to use a robust direct pairwise comparison approach with basic statistics. This decision was made because the dataset we are working with is small and we are thus very limited in the computations we make and the conclusions we can draw from the results. The coefficient of determination and the Pearson correlation coefficient were computed between anatomical measurements of the head and body and physiological measurements and its behaviour on the whole set of horses as well as within the four clusters. These values indicate the body measurements that seem the most related to the behaviour characteristics. High values of the coefficients in our results do not (and cannot) prove any causal link between the anatomical and behavioural measurement. These results only serve as weak indicators of possible connections between them. They are what we can draw from the limited experiments we have performed and should be interpreted accordingly with reservation. They can however serve as guides for further study and more experiments and collected data are needed for any more confident claims about these relationships.

There are too many pairs of measurements to include them all in the paper. For clarity we only show the pairs with the greatest values of the two coefficients, while others are presented in the Appendixes (Supplementary Table S1). We chose two threshold values for the coefficient of determination, using a threshold of 0.3 for coefficients related to the anatomical measurements that were found significant in all the horses and a coefficient of above 0.8 for the measurements significant in separate groups of horses to judge the coefficients in the clusters more harshly due to their smaller size.

**Figure 1:**
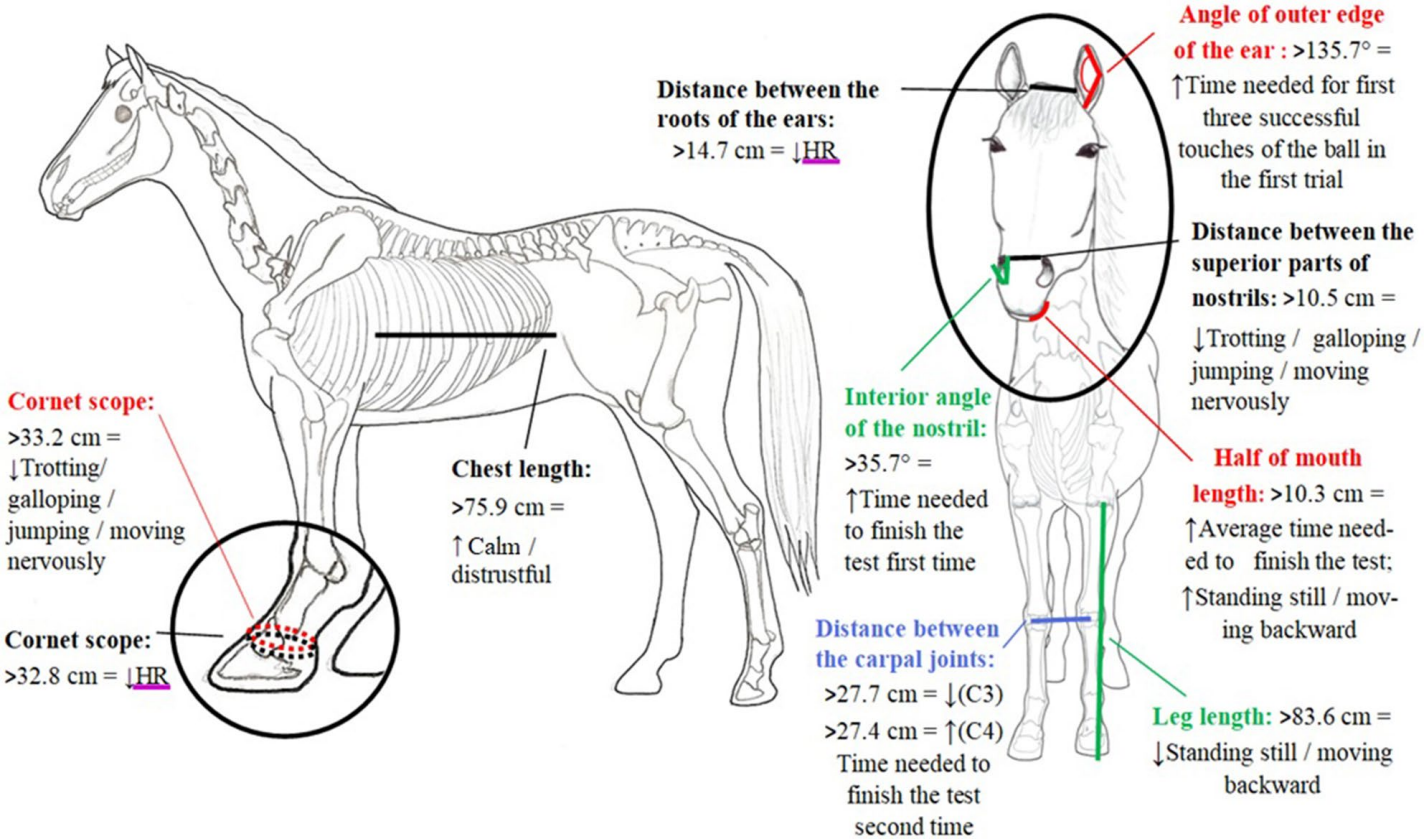
Anatomical measurements with only coefficient of determination for predicting behaviours and heart rate. Black line—all horses, green line—intermediate group, red line—low trainability group, blue line—low fearful group (C3) and low cooperation group (C4), purple line—relates to heart rate. ↓ ↑—relationship between measures and behaviours or heart rate if the anatomical measurement increases.

Regardless of the group an individual horse was allocated to, four anatomical measurements were found to be correlated either with the max heart rate (HR) during the second trial in the HT or calm / distrustful behaviours observed in the FRT or locomotor activity (fast moving forward) performed in the HT (Table 1, Fig. 1). These measures were (1) a cornet scope of front leg (FB12), explaining almost 36% of variability of the max heart rate, (2) distance between the roots of the ears (FH01), explaining 32% of variability of the max heart rate, (3) chest length (FB20), explaining 35% of the variability for behaviours in the FRT, and (4) distance between the superior parts of nostrils (HMP13), explaining 33% of the variability found for behaviours in the HT.

**Table 1:** Anatomical measurements with only significant coefficient of determination for predicting behaviours, with top number presenting the coefficient of determination (degrees of freedom, P-value, F-value) and the bold font number presenting the Pearson correlation coefficient (degrees of freedom, P-value).

Group	Measurement	Target training test			Handling test				Fear reaction test
		Time needed to finish the test first time	Time needed to finish the test second time	Time needed for first three successful touches of the ball in the first trial	Average time needed to finish the test	Fast moving forward	Avoidant	Max HR second trial	Calm / distrustful
All tested horses	Cornet scope of front leg							0.36 (35, 0.64, 0.8)	
								**− 0.60** (33, 0.0001)	
	Chest length								0.35 (35, 0.0006, 6.6)
									**0.59** (33, 0.0004)
	Distance between the roots of the ears							0.32 (35, 0.10, 3.7)	
								**− 0.57** (33, 0.0003)	
	Distance between the superior parts of nostrils					0.33 (35, 0.12, 1.80)			
						**− 0.58** (33, 0.0002)			
Intermediate	Length of a front leg						0.82 (10, 0.0002, 6.8)		
							**− 0.91** (8, 0.0002)		
	Interior angle of the nostril	0.84 (10, 0.0002, 3.0)							
		**0.91** (8, 0.0002)							
Low trainability	Cornet scope of front leg					0.96 (6, 0.005, 46.8)			
						**− 0.98** (4, 0.0006)			
	Half of a mouth length				0.96 (6, 0.05, 17.5)		0.97 (6, 0.004, 53.1)		
					**0.22** (4, 0.0007)		**0.98** (4, 0.0004)		
	Angle of outer edge of the ear			0.97 (6, 0.10, 51.8)					
				**0.98** (4, 0.0003)					
Low fearful	Distance between the carpal joints of the forelegs		0.84 (8, 0.05, 5.8)						
			**− 0.70** (6, 0.05)						
Low cooperation			0.84 (11, 0.19, 16.17)						
			**0.92** (6, 0.00007)

In the horses from the intermediate group (C1 horses) the inferior angle of the nostril (HMP95) explained 84% of the time needed to successfully finish the first trial in the TTT (Table 1, Fig. 1). If the angle was greater, the horse needed more time to successfully finish the trial. The length of a front leg (FB32) explained 82% of the variability in time spent standing still or/and moving backward in the HT. If a horse had longer legs, it spent less time in an inactive state. In the horses from the low trainability group (C2 horses), a cornet scope of front leg (FB12) explained 95% of variability in time spent jumping, trotting, galloping and/or moving nervously in the HT. The correlation was found to be negative, meaning that a horse with a larger scope spent less time performing locomotor activities. A half of a mouth length (FH19) was significantly correlated with two behaviours: an average time an individual horse needed to finish HT and standing still and/or moving backward in the HT, explaining 96% or 97% of their variability, respectively. Horses with a bigger mouth needed more time to finish the test and spent more time in an inactive state. The angle of outer edge of the ear (HMP81) explained 97% of the variability in time the horse needed to finish the first part of the first trial in the TTT, with horses having a bigger angle of the ear needing more time to finish.

In the horses from the low fearful and low cooperation groups, the distance between the carpal joints of the forelegs (FB29) explained 84% of variability in time the horse needed to finish the second trial in the TTT. The correlation was found to be negative in the low fearful group while positive in the low cooperation group. Horses in the low fearful group having higher measurements needed more time while, on the contrary, the horses from low cooperation group needed less time to finish the second trial in the TTT.

### Clustering based on behaviour-related variables

Cluster analysis rendered four groups of horses based on the highest values of silhouette score of behavioural responses and between cluster distance values (Table 2). There were 10 horses (28.6%) in the first group (C1 horses; mean ± SD = 13.10 ± 6.37 years of age), 6 horses (17.1%) in the second group (C2 horses; 13.33 ± 5.09 years of age), 8 horses (22.9%) in the third group (C3 horses; 10.38 ± 3.46 years of age) and 11 horses (31.4%) in the fourth group (C4 horses; 10.55 ± 3.21 years of age). Segmentation of behavioural responses into four groups was used to test whether there was a dependence between sex and the calculated groups (the single stallion was combined with the geldings for this analysis). No sex differences were found (chi-square test; Chi^2^ = 1.29; P = 0.73; degrees of freedom = 3; N = 35; data not shown).

**Table 2:** Calculated average values (AV), standard deviation (SD), F-values and P-values for four groups of horses (C1—C4), derived from behavioural data in the behavioural tests.

Behaviours		C1 (N = 10)		C2 (N = 6)		C3 (N = 8)		C4 (N = 11)		F-value	P-value*
		AV	SD	AV	SD	AV	SD	AV	SD		
Target training test	Time needed to finish the test first time ^l^	**61.00**^**ab**^	**87.28**	**132.00**^**a**^	**103.01**	**30.00**^**b**^	**10.98**	**36.00**^**b**^	**27.67**	3.53	**0.03**
	Time needed to finish the test second time ^l^	**45.00**^**b**^	**29.72**	**238.00**^**a**^	**49.74**	**45.00**^**b**^	**20.64**	**32.00**^**b**^	**21.34**	72.55	** < 0.0001**
	Time needed for first three successful touches of the ball in the first trial ^l^	44.00	90.02	89.00	111.86	16.00	8.01	20.00	12.29	1.73	0.18
	Time needed for second three successful touches of the ball in the first trial ^l^	**17.00**^**ab**^	**22.26**	**43.00**^**a**^	**24.12**	**13.00**^**b**^	**7.46**	**16.00**^**ab**^	**18.23**	3.62	**0.02**
	Time needed for first three successful touches of the ball in the second trial ^l^	**26.00**^**ab**^	**12.10**	**134.00**^**a**^	**111.43**	**24.00**^**ab**^	**12.46**	**17.00**^**b**^	**9.20**	9.93	** < 0.0001**
	Time needed for second three successful touches of the ball in the second trial ^l^	**19.00**^**b**^	**18.74**	**104.00**^**a**^	**120.25**	**21.00**^**b**^	**13.45**	**15.00**^**b**^	**16.77**	4.78	**0.01**
	Attentive/curious ^s^	93.34	12.59	93.32	8.09	96.58	6.40	96.24	3.87	0.38	0.77
Handling test	Average time to finish the tests ^l^	30.00	5.49	77.00	117.01	52.00	57.13	106.00	105.58	1.66	0.20
	Fast moving forward ^s^	1.80	3.74	9.52	15.43	8.39	7.85	9.53	9.51	1.54	0.22
	Avoidant ^s^	0.59	1.86	14.63	34.10	6.17	8.66	19.75	24.12	1.84	0.16
	Attentive/curious ^s^	**56.48**^**a**^	**19.23**	**50.57**^**ab**^	**31.24**	**54.57**^**a**^	**27.10**	**20.25**^**b**^	**13.71**	6.05	**0.002**
	Calm/distrustful ^s^	**34.69**^**b**^	**18.22**	**37.60**^**ab**^	**25.41**	**38.03**^**b**^	**15.58**	**71.19**^**a**^	**24.05**	6.88	**0.001**
Fear reaction test	Fast moving forward ^s^	**8.75**^**b**^	**6.26**	**20.83**^**b**^	**13.75**	**54.96**^**a**^	**12.01**	**20.45**^**b**^	**12.93**	26.35	** < 0.0001**
	Avoidant ^s^	**81.25**^**a**^	**10.09**	**60.00**^**b**^	**17.68**	**24.78**^**c**^	**16.76**	**69.32**^**ab**^	**16.81**	21.84	** < 0.0001**
	Attentive/curious ^s^	2.75	4.63	0.42	1.02	0.63	1.77	7.27	11.86	1.80	0.17
	Calm/distrustful ^s^	**90.00**^**a**^	**4.86**	**82.50**^**ab**^	**8.80**	**58.71**^**b**^	**19.09**	**85.91**^**ab**^	**26.04**	5.25	**0.005**

C1—intermediate horses; C2—low trainability horses; C3—low fearful horses; C4—horses with low cooperation. Pair-wise differences between the four groups are indicated by different superscript letters, with "a" representing the highest AV.

^l^Values represent latencies, measured in seconds (s).

^s^Values represent percentage (%) of the total testing time.

*Pair-wise behavioural results of the horses with statistical significance at 0.05 (p < 0.05/6) in bold.

Groups were tested for statistically significant differences in behavioural measures using the ANOVA test (middle columns of C1-C4 in Table 2) and confirmed with pairwise Student t- tests between groups using the Bonferroni correction (right column of Table 2; see note on correction marked with *). Five of the significant behavioural differences were found in the target training test (TTT), two in the handling test (HT) and three in the fear reaction test (FRT). In the TTT, C2 horses needed the longest to successfully complete the second trial and longer in the first trial when compared to C3 and C4 horses. In the HT, C4 horses were less attentive / curious and more calm / distrustful than the C1 or C3 horses. In the FRT, C3 horses spent the longest time trotting, galloping, jumping and/or moving nervously and consequently they performed the least time standing still and/or moving backward and were less calm / distrustful than C1 horses. Based on these differences in behavioural responses, the groups of horses were labelled as horses with low trainability (C2 horses), low fearful horses (C3 horses), horses with low cooperation (C4 horses), and horses that did not stand out in any of the tests (C1 horses).

Note that this characterisation of the four groups is an interpretation of the differences in the behavioural variables. The clustering method and the statistics comparison only show that there is a division into subgroups which can be claimed to differ in their measurement values with statistical confidence. The characterisation of the differences is effectively authors’ opinion and not a direct result of statistical analysis.

### Comparison of the group segmentation using different assessment methods

To test if Tellington-Jones and Taylor (TJ^12^) and horse personality questionnaire (HPQ) methods identify any differences in horse behaviour, we analysed if groups of horses with distinct behaviour can be formed using these methods’ values alone. We have shown in the previous section that such a split of the horses in our dataset exists. The rationale is that if TJ and HPQ methods capture some information on horse behaviour then we should be able to identify groups with distinct behaviour by using the same clustering approach as before on the TJ and HPQ variables respectively. We ran the clustering algorithm on both sets of variables, clustering the horses into four groups both times. The results of the ANOVA test on the resulting groups are presented in Table 3.

**Table 3:** The ANOVA test for groups of horses that had comparable responses to three behavioural tests, for head characteristics based on Tellington–Jones and Taylor descriptions and for the questionnaire data.

Behaviours		Tellington-Jones and Taylor method		Horse personality questionnaire method	
		F-value	P-value	F-value	P-value
Target training test	Time needed to finish the test (for the) first time^a^	1.19	0.33	0.24	0.87
	Time needed to finish the test second time^a^	1.25	0.31	0.79	0.51
	Time needed for first three successful touches of the ball in the first trial^a^	1.40	0.26	0.66	0.58
	Time needed for second three successful touches of the ball in the first trial^a^	1.28	0.30	1.26	0.31
	Time needed for first three successful touches of the ball in the second trial^a^	1.15	0.34	0.74	0.54
	Time needed for second three successful touches of the ball in the second trial^a^	0.76	0.52	0.15	0.93
	Attentive/curious^b^	0.86	0.47	0.33	0.80
Handling test	Average time to finish the tests^a^	0.94	0.43	0.31	0.81
	Fast moving forward^b^	0.24	0.87	1.27	0.30
	Avoidant^b^	1.46	0.24	0.41	0.75
	Attentive/curious^b^	0.40	0.75	0.40	0.75
	Calm/distrustful^b^	0.44	0.73	0.03	0.99
Fear reaction test	Fast moving forward^b^	0.38	0.77	2.30	0.09
	Avoidant^b^	0.16	0.92	2.04	0.13
	Attentive/curious^b^	2.03	0.13	0.88	0.46
	Calm/distrustful^b^	0.84	0.41	0.43	0.74

^a^Values represent latencies, measured in seconds (s).

^b^Values represent percentage (%) of the total testing time.

No statistically significant differences between groups are found with either method. For only one behavioural feature (Fast moving forward) the groups split on HPQ variables show difference at the significance level of 0.09. For most of the other groups the ANOVA test shows much less significant differences. These results suggest that TJ and HPQ methods do not capture any information useful for discerning horse behaviour. One might try to argue that the problem is in the clustering algorithm and that the information is just encoded in such away in the variables, that the algorithm is too weak to make sense of. Given the relatively simple nature of the TJ and HPQ variables this does not seem likely. Both methods are designed to draw clear indication of horse behaviour, not complex indicators which would need sophisticated processing to interpret.

## Discussion

In this horse study, we identified four general groups of mature Lipizzan horses when exposed to fearful and handling as well as learning situations. Using a standard clustering methodology on behaviour data we identified four groups which were shown to have statistically significant differences in measurements of their behaviour. Through authors’ interpretation of these differences, the groups were designated as “low fearful”, “low cooperation”, “with low trainability” and an “intermediate group where horses did not stand out in their responses”. A statistical comparison of behaviour and physiological measurements was also performed. The results suggest that the size of body and head may affect or even predispose personality traits, which to our knowledge has never been scientifically shown in an animal species. As the dataset is limited, further study is needed on more horses to confirm or disprove these relationships.

From behavioural observations, each individual was assigned to a particular cluster group associated with behaviour responses (including response latencies, limb movements, activities and expressions; Table 4). Our four distinctive clusters may suggest there was enough biological sensitivity to the contexts^27^ but might also reflect different individual experience with humans^28^ since horses were confronted with an unknown person during testing and handling. This factor may have affected horses’ memories of human actions either positively or negatively^29^. There are a number of other potential factors that could have some influence on horse behaviour, such as different training methods and equipment used^18^, fear or novelty of the environment or target^19,23^ or curiosity/motivation^22^, housing conditions^1,27^, and more. To date, the most frequently mentioned categorisation of animal responses to a challenging situation are reactive, proactive (farm animals^30^; horses^4,31^) or intermediate (farm animals^32^). After using a cluster analysis approach, our tests elicited four different categorized behavioural responses as C1, C2, C3 and C4 groups. Based on authors’ interpretation of the groups, group C1 combined intermediate horses, group C2 horses with a low trainability ability, C3 low fearful and C4 low cooperation horses, with the majority of horses being categorised as poorly cooperative, fearful, and having a low level of curiosity. . The minority of horses was categorized as having a low trainability ability (Table 2). This finding may not be surprising, knowing that breeding programs of Lipizzan horses have targeted fast learners.

**Table 4:** Behaviours recorded during the behavioural tests and their definitions.

Category	Behavior	Definition
		Limb movements
Fast moving forward	Trot^a,b^	Movement forward in a two-beat gait, in which diagonally paired feet touch and lift simultaneously
	Gallop^a,b^	Fast four beat gaits
	Jump^a,b^	With mostly hind leg propulsion, moving forward with the forelegs leaving the ground first followed by the hind legs. Jumping can be vertical to clear high obstacles or broad to span ditches or small streams
	Nervous movements^a^	Any individual movement in each direction of any of the four legs that did not have a pattern
Avoidant	Still^a^	The horse has all four hooves on the ground and not moving any of them
	Backward^a^	Movement backward in slower or faster four beat gaits
		Expressions
Attentive/curious	Alert/attentive^a,b^	Rigid stance with the neck elevated and the head oriented toward the object or animal of focus. The ears are held stiffly upright and forward, and the nostrils may be slightly dilated
	Curious^a^^,b^	Stretched neck with movement, eyes and ears faced towards object of interest. Base of the tail is raised above back line, wrinkled lips and movements of nostrils can be seen when sniffing the object
Calm/distrustful	Calm^a^^,c^	Head is low with ears hanging freely at the side of the head. Eyes are open, tail is inactive
	Distrustful^a^^,b^	Head is high with tense neck and ears moving backward and forward. Focus of the head and eyes varies from the point of interest and away from it. Tail is inactive while horse can balk
		Latencies
Finish testing	Time needed to finish the test first and second time in HT, separated	The time when the horse’s first front leg crossed beginning line of the test was recorded and time when its last hind leg crossed the end line. The duration presented time needed for horse to finish test
	Time needed to finish the test first and second time in TTT, separated	The time of the first touch and the last, 6th touch of the ball was recorded. The duration presented time needed for horse to finish test
Touching a ball	Time needed for the first three successful touches of the ball in the first and second trial, separated	The time of the first and third touch of the ball was recorded. The duration presented time needed for horse to partially finish test
	Time needed for the second three successful touches of the ball in the first and second trial, separated	The time of the fourth and sixth consecutive touch of the ball was recorded. The duration presented time needed for horse to partially finish test

*TTT* target raining test, *HT* handling test.

^a^Duration.

^b^McDonnell^48^.

^c^Draaisma^34^.

^d^Values represent percentage (%) of the total testing time.

Correlation analysis found nine characteristics of body (n = 4) and head (n = 5) to be indicative of behaviour and heart rate during test (Table 1, Fig. 1). These results show some promise that, by using a larger sample size, a connection between physiological characteristics and the behaviour types could be confirmed. It remains unclear here to what extent our horses were under the influence of cardiovascular fitness, because although activity levels were low during behavioural testing, horses were of different ages and had different levels of previous training or exercise.

In addition, we provide initial evidence that the anecdotal beliefs of an association between personality traits on one hand and specific body and head measures as well as cardiovascular activity on the other hand exist. Although it could be argued that age was a confounding factor, since age and body measurements are related, the age criterion was breed-related, since a five-year-old Lipizzan horse is mature and full-grown at this age and therefore does not change significantly in the following years^33^. Although the shape and size of the horse's skull may vary by sex^33^, this factor did not prove to be influential in the formation of personality groups in this study, so we were able to exclude the sex effect from further analysis. While respecting the relative weakness of evidence available so far, we offer a possible interpretation of these differing characteristics.

Out of the nine mentioned characteristics, four related to all the horses tested, two on the head (i.e., distances between the roots of the ears and between the superior parts of the nostrils) and two on the body (cornet scope of front leg and chest length). When the chest was longer, horses showed more calm / distrustful emotional reaction in the fear reaction test situation that may, according to its specifics^18^, present most risk to riders and handlers of horses. This indicates that horse breeders may have difficulties building trustworthy relationships with horses with longer backs since trust is essential during daily handling routines^3^ in order to prevent injuries of both rider and a horse^31^.

We do not rule out the possibility that horses with longer backs experienced higher levels of discomfort or even pain because of their anatomic characteristics, although this is pure speculation because there are no reliable and objective data to support this claim^11^. In addition, our horses were not considered lame during testing and no obvious signs of pain were noted (e.g., unusual posture, shifting weight from one leg to the other, muscle tremors, abnormal sweating, lying down more frequently than usual, decreased appetite, signs of injury). Because we did not restrict a horse's movements in the fear reaction test, or only when a horse decided to turn its back to the handler, the observed distrust and calmness (i.e., when a horse was unwilling to respond to stimuli and seemed to withdraw into itself and shut down completely in response to a stimulus^34^) may be related to higher levels of fear^20,21^ or previous bad experiences related to the test situation^34^ or with people^28^.

When distances between the roots of the ear and cornet scope of front leg were greater, heart rate decreased, however, in the second trial of the handling test only. This may result from a greater distance between the nostrils leading to the observed decrease in physical (locomotor) activity. Assuming that completing the handling test with lower activity is a sign of a greater stimulus control, ease to handle^23^, and a lower level of fear^19,23^, our results imply that Lipizzan horses with wider heads and greater cornet scopes (i.e., strong legs) are calmer and more trustworthy, particularly after the second trial of testing.

To our knowledge this is the first evidence of a link between HR and anatomical characteristics of a horse’s body and head in a human handling context. Previously, Górecka et al.^7^ showed a lack of correlation between heart rate measures in a startle context and hair whorl height. Considering the results associated with all the horses, we suggest that the chest length (i.e., longer back) and distance between nostrils (i.e., wider muzzle) may be predictive of future level of trustworthiness in Lipizzan horses.

From a selection point of view^35^ and in conjunction with our findings, Lipizzan horses having strong legs, a wide head and a short back would be preferred since they were found to be calmer and easier to train with a lower heart rate. We therefore suggest them to be safer and less time consuming to train. According to Grandin and Deesing^6^, learning ability, memory, novelty seeking, activity level, fearfulness and sociability all show some degree of genetic influence. Therefore, for future studies with the objective of providing more robust breeding guidelines, we suggest to compare at the genome level clearly defined phenotypic groups which can provide the information about the underlying genetic variants.

The clustering into four behavioural groups was not associated with either the head characteristics described by TJ or results gathered by a HPQ that was filled-in by the horse trainers or owners. This means that after horses were clustered using these two methods, these groups did not show consistent differences in behaviour, implying that these methods do not have predictive value for traits such as fearfulness, handling/cooperation and trainability ability. Although TJ^9^ provided some insights into the characteristics of a horse’s head linked to specific horse personality traits, the use of this method needs profound experience with the visual conformation scoring. Furthermore, the description of personality traits is too broad to be precisely analysed and unreliably scored across assessors as such assessment may depend on how good an assessor knows a horse and on interpretation of an individual trait^36,37^.

Similarly, Seaman et al.^38^ reported no relationship between the responses in their behavioural tests and the questionnaire ratings given by the farm team leader. To minimize the risk of subjectivity by the human respondents in the questionnaire, a larger number of respondents per horse and more complex statistical analysis are suggested to be used^39^. In our study, unfortunately, we were able to collect only one questionnaire per horse. We also did not find any correlation between behavioural responses and hair swirls position, an association suggested previously^7,8,40^.

Although some attempts have been made in other species (pigs and cattle:^6^; dogs:^15,16^, including humans^41^, all suggestions of an association between personality and anatomical characteristics are to date scientifically unproven. However, there is one study worth mentioning and that is Belyaev's world-famous domestication and selection experiment on foxes, in which a relationship between the personality trait tameness and anatomy was suggested, but only with a weak tendency^42^. They found that the tamed foxes tended to be slightly larger, their skulls tended to be smaller, and their muzzles tended to be shorter and wider than those of the control foxes.

In this study, the pilot results show the first rigorous evaluation of a scientific association between behaviour that assigns an individual to a specific personality category, physiological response and anatomy in horses. Since standardised behavioural tests for identifying Lipizzaners for specific use are not available in Slovenia or worldwide, there is a clear need for research into approaches for complex evaluation of horse personality. First of all, the selection process is lengthy, and if the phenotype of the horse does not match the chosen task, this often leads to various health problems (e.g. problems in movement and subsequently back problems). We therefore believe that the development of more objective methods is necessary.

We suggest that anatomical characteristics found in Lipizzan horses give a reliable and objective measure to define personality traits of an unknown horse. Our conclusions are based on a small number of animals, therefore it is important to conduct more work to ensure reliability of the method and to generalise the interpretation of the results to a wider cohort of Lipizzan horses. We believe that our study serves as a foundation for future research on physio-anatomical characteristics of horse personality in order to find individuals best suited for a specific use and thus improve handler safety and horse welfare.

## Materials and methods

### Ethical statement

All procedures with animals were in accordance with the principals of the 3Rs and were performed according to the legislation on animal experimentation in Slovenia. The experimental protocols were approved by the animal-welfare body at the Department of Animal Science that is a member of the Ethical Committee of the Administration of the Republic of Slovenia for Food Safety, Veterinary Sector and Plant Protection (UVHVVR).

### Animals

The study involved 35 Lipizzan horses (n = 17 mares, n = 1 stallion, n = 17 geldings) that originated from five horse facilities in Slovenia. The horses were five years old or older, they were ridden (trained to a saddle, performed changes in direction and speed under command by using classical, and/or traditional English style riding principles) and were healthy (i.e. internal body temperature, measured rectally daily with a digital thermometer, below 39 °C; no previous medical problems including musculoskeletal disorders). Following the riding principles used, horses were not introduced to marker training or targeting. All horses were kept individually in boxes during the night and in groups on pastures or paddocks during the day. They were offered fresh hay ad libitum and were mainly fed a barley-oat mixture, the amount and composition of which were adjusted to the horse's weight, size, and daily workload.

Most horses had been purchased as yearlings, therefore we were not able to collect data on their early experience with humans, although it is believed that early formation of the foal-human relationship influences an animal’s personality traits such as fearfulness and trainability^6^ and thus can shape responses later in life. Many of them had an unknown number of previous owners and riders with different riding expertise. Our horses were used for sport or as leisure horses with different daily use (lunging or/and riding under saddle, leisure activities).

### Data collection

On each test day, physical condition (lameness, body condition, eye and nose discharge, body injuries) was examined before testing to determine possible pain or discomfort. In one case, a horse injured itself and testing had to be performed on another day. Physiological measures (heart rate (HR) and heart rate variability (HRV)) were first taken during rest while horses were stalled in an individual box, which was their familiar environment. They were loose in the box and without halters. These physiological measurements were collected once daily and repeated for five consecutive days. Behavioural tests always applied in the same following order; the HT, the TTT, and the FRT. We took into account that the horses were first tested with less intimidating stimuli (two umbrellas and a yellow foam ball represented new visual stimuli that did not involve movement) before performing the FRT test, in which the bag represented a moving new visual stimulus and an auditory stimulus that, as such, may be perceived as more intimidating. During these tests, the horses’ HR was also monitored. Next, we gathered anatomical measurements of head and body while the horse was on a flat surface. In addition, the HPQ was sent to the owners/trainers of individual horses.

### Behavioural tests

To test the personality of a horse, three behaviour tests were conducted by a single handler unknown to the horses: HT, TTT and FRT. Tests were carried out consecutively in this order, and were repeated twice with two days between repetitions. Our behavioural tests were adapted from the descriptions published in previous studies (reactivity test or novel object test: Górecka et al.^7^; handling test or response to a person: Seaman et al.^38^; arena test: Seaman et al.^38^; ridden work tests:^43^; problem solving test:^21^).

Briefly, the behavioural tests were conducted inside a testing field (15 m × 6 m), which was located in a fenced area familiar to the horse near the home horse facility. In the HT, two identical open umbrellas (85 cm) were positioned 5 m apart so that they formed a passage through which the handler led an individual horse. Prior to the start of each test, horse was allowed 10-min adaptation period that was followed by the beginning of the test after the beginning line was passed by horses’ first front hoof and ended after the horse's last hind hoof touched the ground 5 m from the umbrellas. The 10-min adaptation periods were set taking into account that horses are animals that need to adapt quickly to constantly changing challenges^44^ and because no obvious behavioural problems were observed between the periods in our pilot study with 5 horses^45^. In the FRT, the handler positioned the horse 5 m from an assistant who held a whip (65 cm long) with an attached blue and white plastic bag (40 × 30 cm). The assistant then waved it in line of his body and facing the horse, using a fast, smooth and firm movement of figure ∞ for 20 s.

In the TTT, horses were expected to touch a yellow foam ball (10 cm diameter, positioned 10 cm from horses’ muzzle) at the end of a stick (50 cm long) 6 times while standing one meter away from a handler. They had no prior training or experience of this object. For the first three successful touches, a horse received a carrot cube, while for second three touches (ball unchangingly moved left, right and back left from horses’ muzzle while maintaining 10 cm distance between ball and muzzle) no reward was given. The decision to observe responses that are no longer reinforced after a discriminative stimulus, the process known as extinction^46^, is based on the results of Valenchon et al.^47^, who showed that the most fearful horses were most resistant to extinction during the backward task. The videos of each behavioural test were stored on a computer and analysed by a single person, who did not know the tested horses, according to a predefined ethogram (Table 4) using the programme VLC media player. This final ethogram is a result of our pilot observations on 5 horses^45^.

### Physiological measures

HR(V) during rest and during behavioural tests were recorded using Equine H7 heart rate sensor electrode base set and a receiver Polar V800 heart rate monitor (Helsinki, Finland) with Bluetooth Smart® wireless technology. Automatic calibration was performed twice, and the average of the two calibration factors was used. Contact electrodes were attached to a non-standard elastic belt, made for the purpose, and electrodes were placed to correct positions on a horse girth area. The contact between the rubber electrode areas and the horse’s skin was optimized by using contact gel on the electrode areas. We placed a piece of furnishing foam beneath the electrodes and the elastic belt. Prior to testing, we let the horses get used to an elastic belt for 10 min.

The resting values HR and HRV were recorded for the duration of about 60 min (from 45 to 90 min). The measurements were taken between 5:30 a.m. and 12 a.m., before the horses started with work or training, or from 6 p.m. to 8 p.m. after the horses went to rest. While measuring basal HR, owners and personnel were asked not to enter the stable to ensure a calm environment for the horses. We also asked owners of the horses to train their horses with fairly easy training sessions (i.e. avoid all day exercise) during the five days when the measurements were taken, as a hard training session the previous day can cause elevated heart rate at rest^49^. The analysis of HR data from behavioural tests was made in the Polar Flow app, where we recorded the highest HR measurement. Basal HR data recordings were analysed in program Kubios HRV, where the most representative sample (i.e. consistent RR fluctuation during 5-day recordings; not standing out with an increase/decrease) of 14 min within one-hour recordings was analysed while artifact correction was set to medium and frequency bands were defined as very low frequency from 0 to 0.04 Hz, low frequency from 0.04 to 0.13 and high frequency from 0.13 to 0.4 Hz. HR data from the behavioural tests were not corrected for possible baseline differences in HR.

Body temperature was measured rectally with a digital thermometer after HR measurements, between 5:40 a.m. and 1 p.m. If the body temperature exceeded 39 °C, that horse was not included in the study on that day.

### Anatomical measurements

**Figure 2:**
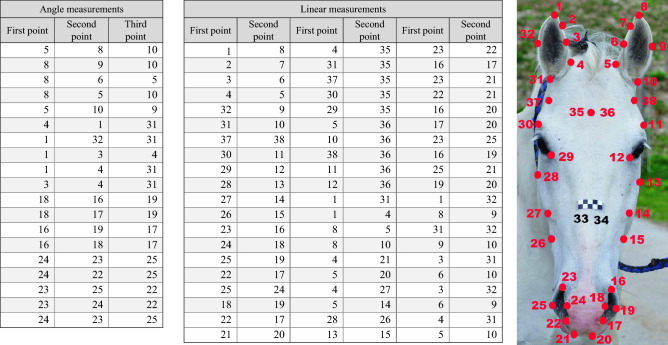
Detailed description of measurements, extracted from the CoordGen8 computer program. Picture of the horse front head profile on the right represents how landmarks were positioned on the horse's head picture. Each landmark had an individual number that was consistent throughout all 35 horse pictures. Black numbers 33 and 34 were markers for unit of 1 cm (distance between them was 1 cm). Table on the left represents measurements that were extracted from landmarks. Angle measurements represent angles, measured between 3 landmark points (first point, second point and third point) marked on the horse’s head profile picture on the right, while linear measurements were actual distances between two landmark points (first point and second point) marked on the horse’s head profile picture on the right.

Anatomical measurements were collected using three different approaches. First, according to the instructions given by Tellington-Jones and Taylor^9^, we took a set of head pictures (resolution: 300 of pixels per inch) with camera Nikon D90 (12.3—megapixel digital single-lens reflex camera) of each horse where the horse’s front and left head profile was visible. Interpretation of pictures was done by following descriptions from the book of Tellington-Jones and Taylor^9^. Second, we additionally obtained a photo of the front profile by the same procedure as described above, with an addition of a ruler that consisted of 1 cm × 1 cm square blocks (2 cm × 5 cm, printed on self-adhesive white paper), that was attached to the front view of the horse's head, on the line of a lower end of the facial crest (Fig. 2).

**Figure 3:**
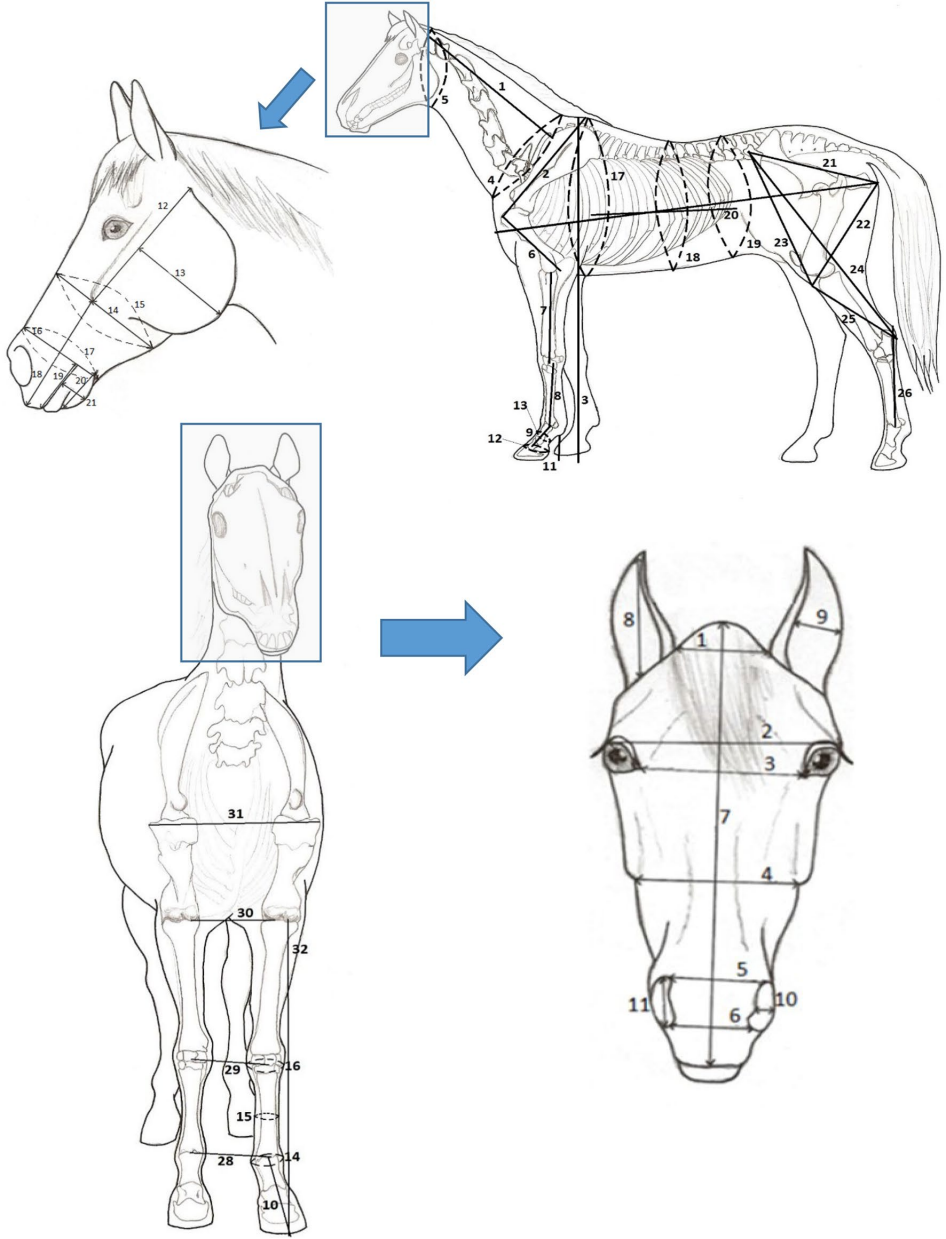
Detailed description of measurements, measured on the horse body and head. The horse body drawing represents important skeletal structures that were used during measurement as a reference point and we were able to locate them by using a palpating technique. The head drawing shows major anatomical characteristics of the horse’s head that were also used as reference points to ensure comparable measurements between different horses.

Pictures from this approach were used to measure distances on the horse’s head using a tpsDig232 program and CoordGen8 software. A total of 88 distances and angles extracted once from a single person using the CoordGen8 program were converted to cm (Fig. 2). Final, head anatomical characteristics were measured with a sartorial meter (Fig. 3). In total, 19 head measurements were gathered on the horses’ left or front sides of the head, and two head scope measurements. In addition to head measurements, the sartorial meter (a 2.5 m bendable meter with metric units) was used to collect 32 body measurements on the horses' left or front sides of the body (with the exception of scopes) (Fig. 3). Measurements with a sartorial meter were done twice in two days.

### Questionnaire for horse trainers/owners

Horse owners or trainers received a paper-and-pencil HPQ, adapted from Lloyd et al.^50^ (Supplementary Table S2) that consisted of a list of 30 adjectives with a number scale from one to seven. The HPQ was filled out by marking the appropriate number based on a list of adjective definitions. The value of one meant complete absence of a specific behaviour, the value of seven meant full expression of it, while number four represented an average value.

### Statistical analysis

To reveal individual differences in behavioural characteristics of the horses, data from behavioural tests were used for segmentation of the horses into groups using standard clustering methodology. The K-means algorithm was applied and Euclidean distance (computed by the Pythagorean formula) was used as distance measure. We used the implementation that is part of the Orange software package (version 2.7). To assess quality of the segmentation of horses into groups, the silhouette score and between cluster distance was computed. In order to identify the characteristics in which differences were statistically significant between the clusters, we used the standard Student T-test for the analysis of clustering into two groups and the ANOVA test for clustering into four groups. To gain further insight into the differences between clusters we used the Bonferroni correction for the pairwise T-tests. Next, we divided all of the horses into 4 groups based on the Tellington-Jones and Taylor values and HPQ values using the same k-means methodology. Having identified which horses belonged to which groups in the next step, used Pearson’s chi-squared test, we investigated if those groups differed according to behavioural test results. The results did not show a dependence between the clusters, which meant the groups are different. Furthermore, we compared the groups using the ANOVA test on the behavioural characteristics of the horses.

Having established the 4 characteristically distinct groupings of horses based on behaviour, we tested the predictive power of anatomical characteristics for classification into these groups. We also computed the coefficient of determination (R2) and Pearson correlation coefficient between the anatomical, physiological and behavioural characteristics and HR responses in behavioural tests for all the horses and for each of the groups separately in order to directly test the relations between the two types of measurements. The two coefficients indicate the predictive value of each individual anatomical measurement for each individual behavioural characteristic independently. This gives a more fine-grained and robust indication of the relations, which are independent of modelling technique and clustering.

## Supplementary Information

Supplementary Table 1.

Supplementary Table 2.

## Data Availability

The datasets gathered during the current study are available on request from the corresponding author.
