# Supplementary material for: Relationship between anatomical characteristics and personality traits in Lipizzan horses
Source: Sci Rep. 2022 Jul 23;12:12618. doi: 10.1038/s41598-022-16627-z (PMC9308772; doi:10.1038/s41598-022-16627-z)
Supplement: Supplementary file 2 — Supplementary Table 2. [file 41598_2022_16627_MOESM2_ESM.pdf]

Supplementary Table S2: Behavioural definitions of personality adjectives derived from Lloyd et al. (50) for the use in horse personality questionnaire

Personality adjective	Behavioural definition
Active	Moves around a lot, does not like being still for long
Aggressive	Causes harm or potential harm to other individuals, both horse and human
Apprehensive	Seems to be anxious about everything, fears or avoids any kind of risk
Confident	Behaves in a positive, assured manner, not restrained, tentative
Curious	Readily explores new situations
Eccentric	Shows stereotypes, unusual mannerisms and exaggerated behavior
Effective	Gets own way, can control others, fairly dominant individual
Equable	Reacts to others in an even, calm way; not easily disturbed
Excitable	Over reacts to any change, easily excited, highly strung
Fearful	Retreats readily from others or from outside disturbances
Insecure	Hesitates to act alone; seeks reassurance from others
Irritable	Reacts negatively with little provocation
Motherly	Provides warm receptive secure base for others, is tender and caring
Opportunistic	Seizes a chance as soon as it arises
Permissive	Could, but does not interfere with behaviour of others
Playful	Initiates play and joins in when play is solicited
Popular	Sought out as a companion by others
Protective	Prevents harm or possible harm to others
Slow	Moves and rests in a relaxed manner, moves slowly and deliberately, not easily hurried
Sociable	Seeks companionship of others
Solitary	Spends a lot of time alone by choice
Subordinate	Gives in readily to others, submits easily and does not put up a fight to defend self
Strong	Depends upon sturdiness and muscular strength
Tense	Shows restraint in posture and movement; carries the body stiffly, which suggests a shrinking tendency, as if to pull back and be less conspicuous
Understanding	Responds in a discriminating and appropriate manner to the behaviour of others
Suspicious	Does not trust others readily (human and horse), trusts few individuals
Reliable	Can be trusted to do things or behaves well might also be considered a safe horse to be with
Hardworking	Keen to do well, behaves well during ‘work’, and concentrates on what it is being asked to do
Stubborn	Does not give in easily, not very cooperative
Intelligent	Learns new things easily/fast benefits from mental stimulation
